# Tacrolimus ameliorates pulmonary hypertension associated with pulmonary fibrosis via modulation of vascular remodeling

**DOI:** 10.3389/fphar.2026.1880903

**Published:** 2026-10-26

**Authors:** Xueze Liu, Qi Chen, Yayue Hu, Zhigang Liu, Zhongyi Yang, Jiayang Yu, Huihui Li, Ruxuan Chen, Chi Shao, Shiyang Wang, Cheng Yang, Xiaohe Li, Hui Huang

**Affiliations:** 1 State Key Laboratory of Medicinal Chemical Biology, College of Pharmacy, Nankai-Baiyang Joint R&D Center for Basic and Translational Medicine of Organ Fibroproliferation, Nankai University, Tianjin, China; 2 Department of Pulmonary and Critical Care Medicine, Peking Union Medical College Hospital, Chinese Academy of Medical Sciences and Peking Union Medical College, Beijing, China; 3 Tianjin Key Laboratory of Molecular Drug Research, Tianjin International Joint Academy of Biomedicine, Tianjin, China

**Keywords:** idiopathic pulmonary fibrosis, MAPK pathway, PI3K/Akt pathway, pulmonary hypertension, tacrolimus, vascular remodeling

## Abstract

**Introduction:**

Idiopathic pulmonary fibrosis (IPF) is frequently complicated by pulmonary hypertension (IPF-PH), a severe condition that worsens symptoms, reduces exercise capacity, and shortens survival. Current anti-fibrotic and PH-targeted therapies show limited efficacy in IPF-PH, and the therapeutic role of tacrolimus in this composite disease remains underexplored.

**Methods:**

A bleomycin-induced IPF-PH rat model was established. Rats were randomized into control, bleomycin model, treprostinil (positive control), and tacrolimus (0.05 mg/kg/day, subcutaneous, 21 days) groups. Right ventricular systolic pressure (RVSP), echocardiography, right ventricular and lung histology, immunohistochemistry, qRT-PCR, and Western blot were assessed. In vitro, C166 endothelial cells and primary rat pulmonary arterial endothelial cells were stimulated with bleomycin with or without tacrolimus; MOVAS smooth muscle cells were stimulated with TGF-β1 with or without tacrolimus. Endpoints included cell viability, tube formation, ROS, SOD/GSH, apoptosis, proliferation/migration, and MAPK/PI3K/AKT signaling.

**Results:**

Tacrolimus reduced RVSP, improved PAT/PET and right heart function, attenuated right ventricular hypertrophy and pulmonary fibrosis, and decreased vascular wall thickness and α-SMA expression. It also downregulated fibrosis markers Fn1 and Col1a1. In endothelial cells, tacrolimus restored P-VEGFR2/VEGFR2 and VE-cadherin expression, promoted tube formation, reduced apoptosis (Bax/Bcl-2, cleaved caspase-9/3), and alleviated oxidative stress (ROS, SOD/GSH, Keap1/HO-1). In smooth muscle cells, tacrolimus inhibited TGF-β1-induced proliferation and migration and reduced Ki67 expression. Mechanistically, tacrolimus suppressed MAPK (JNK, ERK, p38) and PI3K/AKT pathway activation *in vivo* and *in vitro*.

**Discussion:**

Tacrolimus ameliorates bleomycin-induced IPF-PH through vascular remodeling-oriented, multi-target effects, including endothelial protection, suppression of oxidative stress and apoptosis, and inhibition of smooth muscle proliferation, partly via MAPK and PI3K/AKT signaling. These findings support tacrolimus as a potential therapeutic candidate for IPF-PH, especially in patients with prominent vascular remodeling, while highlighting the need for further dose-response, target-specific, immunomodulatory, and delivery optimization studies before clinical translation.

## 1 Introduction

Pulmonary hypertension (PH) is a severe cardiopulmonary vascular syndrome characterized by markedly elevated pulmonary arterial pressure and vascular remodeling (Zhang et al., 2023). These pathological changes collectively lead to sustained right ventricular overload, ultimately resulting in right ventricular hypertrophy (RVH) (Zhang et al., 2024). Idiopathic pulmonary fibrosis (IPF) is a chronic, progressive and fibrotic form of interstitial pneumonia of unknown etiology, clinically marked by progressive decline in lung function and worsening dyspnea (Porter et al., 2021). PH is a common and serious complication of IPF (Gall et al., 2017). Its onset and progression not only significantly exacerbate physical impairment and reduce quality of life but also markedly shorten survival (Waxman et al., 2022). According to statistics, approximately 8%–15% of IPF patients already have a mean pulmonary arterial pressure ≥25 mmHg at initial diagnosis. This proportion rises to 30%–50% in advanced stages and exceeds 60% in end-stage disease (Humbert et al., 2023).

Currently, although anti-fibrotic agents (e.g., pirfenidone, nintedanib, and nerandomilast) can slow the lung function decline in IPF, there is insufficient evidence to suggest their ability to delay the development of IPF-associated PH. Moreover, some studies suggest that nintedanib may even be associated with PH progression in IPF-PH patients (Lacedonia et al., 2022). Among commonly used therapies in PH, only inhaled treprostinil has shown potential in preliminary clinical studies to slow lung function decline and improve exercise tolerance (Nathan, 2023). In contrast, other PH-targeted drugs, such as endothelin receptor antagonists and soluble guanylate cyclase stimulators, have failed in clinical trials for IPF-PH due to lack of efficacy or increased mortality in the treatment groups.

The pathogenesis of IPF-PH is complex and not fully understood, which is driven by a combination of factors including pulmonary interstitial fibrosis, hypoxia and hypoventilation, inflammatory responses, vascular endothelial dysfunction, immune interactions, abnormal cytokine expression, and genetic factors (Rajagopal et al., 2021). Current research focuses primarily on pulmonary vasoconstriction and remodeling: interstitial fibrosis in IPF patients leads to thickening of the medial and intimal layers of pulmonary vessels, resulting in vascular constriction (Barratt and Millar, 2014). This is partly due to mechanical compression of peripheral or central pulmonary vessels by collagen fibers and vascular remodeling driven by excessive proliferation of fibroblasts and smooth muscle cells (Nakamura et al., 2025). Vascular endothelial cells (ECs) play a central role in the vascular remodeling process, and their dysfunction and phenotypic transformation are key drivers of PH progression (Fan et al., 2025). Under healthy conditions, pulmonary endothelial cells are central to maintaining vascular homeostasis by regulating vascular tone, inflammation, permeability, coagulation, and smooth muscle cell growth (Xing et al., 2024). In IPF-PH patients, this balance is disrupted. Hypoxia, oxidative stress, inflammatory mediators, and mechanical injury disrupt endothelial function, leading to reduced nitric oxide and prostacyclin signaling and increased production of vasoconstrictive mediators such as endothelin-1. Overexpression of substances such as endothelin-1 promotes abnormal proliferation of pulmonary arterial smooth muscle cells, leading to sustained pulmonary vasoconstriction and increased resistance, which lays the groundwork for subsequent structural remodeling (Yen et al., 2022). Furthermore, excessive generation of reactive oxygen species (ROS) in pulmonary vessels is a major cause of endothelial cell injury and dysfunction (Zhao et al., 2024). ROS can directly damage cellular structure and function and even induce endothelial cell apoptosis, thereby impairing the ability of endothelial cells to maintain vascular homeostasis and ultimately exacerbating vasoconstriction and elevated vascular resistance. Endothelial oxidative injury and apoptosis may further impair endothelial repair and angiogenic capacity, thereby aggravating pulmonary vascular remodeling.

Tacrolimus (TAC, also known as FK506), a macrolide antibiotic derivative, is a potent immunosuppressant that primarily exerts immunomodulatory effects by inhibiting T-cell activation (Hooks, 1994). Current research suggests that tacrolimus may alleviate both bleomycin-induced pulmonary fibrosis (Liu et al., 2022; Liu et al., 2024) and monocrotaline-induced pulmonary hypertension (Peng et al., 2022). However, its role in IPF-PH remains insufficiently understood and lacks systematic investigation. Therefore, this study aims to explore the therapeutic efficacy of tacrolimus in a bleomycin-induced IPF-PH model and preliminarily elucidate its underlying mechanisms of action.

## 2 Experimental materials and methods

### 2.1 Animal models and treatment approaches

Male Sprague Dawley (SD) rats, weighing approximately 200 g, were purchased from Beijing Vital River Laboratory Animal Technology Co., Ltd. All animal experiments were approved and conducted under the guidelines of the Institutional Animal Care and Use Committee (IACUC) of Nankai University (Permit No. 2024-SYDWLL-000444). The animals were housed under a 12-h light/dark cycle with *ad libitum* access to food and water at the Animal Experiment Center of Nankai University. After acclimatization under identical conditions, the rats were randomly divided into four groups (n = 5 per group): control (CTL), model (BLM), Treprostinil (TRE), and Tacrolimus (TAC).

Following adaptation, the experimental model was established. Rats were anesthetized with 1.5%–2.0% isoflurane gas (Baxter Healthcare Corp., R510–22, United States). After anesthesia, the animals were secured, and the surgical area was disinfected with alcohol. A midline incision was made in the neck, and the surrounding muscle tissue was dissected to expose the trachea. An insulin injection needle was then inserted into the trachea near the ribs for intratracheal instillation of bleomycin (970,592, Tokyo, Japan) at a dose of 5 U/kg. The control group received an equal volume of sterile saline. One week after modeling, drug administration commenced. The CTL and BLM groups received daily subcutaneous injections of an equal volume of vehicle (10% DMSO + 90% saline) in the dorsocervical region. The TAC group received daily subcutaneous injections of tacrolimus (0.05 mg/kg, dissolved in the same vehicle) in the dorsocervical region. Treprostinil was administered as a positive control via daily intranasal instillation at a dose of 11.5 mg/kg. The treatment period lasted for 21 days. On day 21, all rats were anesthetized. Relevant evaluation indices were assessed, and tissue samples were collected for subsequent analysis.

### 2.2 Echocardiography analysis

After anesthesia was induced in rats with 1.5%–2.0% isoflurane gas (Baxter Healthcare Corp., R510–22, United States), echocardiographic images of the right ventricle were subsequently acquired using the VisualSonics high-resolution Vevo 2100 system (VisualSonics Inc., Canada). Data were collected to evaluate cardiac function, with parameters including pulmonary artery acceleration time (PAT), pulmonary ejection time (PET), and the calculated ratio (PAT/PET).

### 2.3 Measurement of right ventricular systolic pressure

Right ventricular systolic pressure (RVSP) was measured in mice anaesthetised with 2.5% tribromoethanol using a BL-4220 Bio-Signal Acquisition System (Taimeng, Chengdu, China). The thoracic cavity was quickly opened, and the heart was gently stabilized. A primed needle was then advanced at a 40° angle for 1 cm into the right ventricle. Pressure signals were recorded during waveform stability (≥0 cardiac cycles) before the needle was withdrawn.1

### 2.4 Histological analysis

Lung and heart tissues were fixed in 4% paraformaldehyde for at least 48 h, followed by dehydration and embedding in paraffin. After embedding, the tissues were sectioned at a thickness of 4 μm. The sections were deparaffinized in xylene and rehydrated through a graded alcohol series (xylene, twice for 8 min each; 95% ethanol, 3 min; 85% ethanol, 3 min; 70% ethanol, 3 min; H_2_O, 5 min). Lung tissue sections were stained with Hematoxylin and Eosin (H&E) (Solarbio, G1120, China), Masson’s trichrome stain (Solarbio, G1340, China), and Sirius Red stain (Solarbio, G1473, China). Heart tissue sections were stained with H&E to observe pathological changes in the lung and heart tissues. All procedures were performed strictly according to the manufacturer’s instructions. The images were semi-quantitatively analyzed using ImageJ.

### 2.5 Cell culture and treatment

Vascular endothelial cells (C166) were purchased from iCell Bioscience Inc. (iCell-m118). When the cells reached 70%–80% confluence, they were stimulated with bleomycin and tacrolimus. After 24 h of stimulation, relevant indicators were assessed. Vascular smooth muscle cells (MOVAS) were obtained from Wuhan Pricella Biotechnology Co., Ltd. When the cells reached 70%–80% confluence, they were stimulated with TGF-β1 and tacrolimus, and relevant indicators were evaluated after 24 h of stimulation. All cells were cultured in high-glucose DMEM (Solarbio, Beijing, China) supplemented with 10% fetal bovine serum (FBS, ExCell Bio, China) and 1% penicillin. The cultures were maintained in a humidified incubator at 37 °C with 5% CO_2_. Primary rat pulmonary arterial endothelial cells (PAECs) were purchased from Jinyuan Biotechnology (JY-J396). When the cells reached 70%–80% confluence, they were stimulated with bleomycin and Tacrolimus.

### 2.6 CCK-8 assay

Cells were seeded into 96-well plates and incubated with the designated drugs. Subsequently, CCK-8 reagent (Dojindo, CK04, Japan) was added equally to each well. After incubating the cells for 2 h, the optical density (OD) at 450 nm was measured using a microplate reader (Bio-Rad, Hercules, United States).

### 2.7 RNA extraction and real-time PCR

Total RNA was extracted from cells or lung tissue using Trizol reagent (Invitrogen, 10,296,028, China). The RNA was then reverse transcribed into cDNA with a reverse transcription kit (Tiangen, KR118, China) and subsequently subjected to real-time quantitative PCR (Yeasen, 11198ES08, China). All steps and experimental conditions were performed according to the manufacturer’s instructions. The primers used in this study are listed in Table 1.

**TABLE 1:** Primers for QRT-PCR.

Gene	Forward primer sequence	Reverse primer sequence
R-Gapdh	GTC​GGT​GTG​AAC​GGA​TTT​G	TCC​CAT​TCT​CAG​CCT​TGA​C
R-Fn1	CCA​TCA​CGC​CGA​GCA​TTC​T	TCT​CCT​CTC​TGC​CAT​TAA​CAG​C
R-Col1a1	ATC​AGC​CCA​AAC​CCC​AAG​GAG​A	CGC​AGG​AAG​GTC​AGC​TGG​ATA​G
R-Bax	GAC​TCC​CCC​CGA​GAG​GTC​TT	ACA​GGG​CCT​TGA​GCA​CCA​GTT
R-Bcl2	GAG​GGG​CTA​CGA​GTG​GGA​TA	TCA​AAC​AGA​GGT​CGC​ATG​CT
R-Keap1	CAC​TTC​GGG​GAG​GAG​GAG​TT	GGG​CAG​TCG​TAT​TTG​ACC​CA
R-Hmox1	TTT​TCA​CCT​TCC​CGA​GCA​T	GCC​TCT​TCT​GTC​ACC​CTG​T
M-Gapdh	TGT​GTC​CGT​CGT​GGA​TCT​GA	CCT​GCT​TCA​CCA​CCT​TCT​TG
M-Bax	CCG​AGA​TCG​TCT​TCA​GCA​AA	GAG​GTC​AGG​ATG​GCA​CAG​AA
M- Bcl2	TGA​GGA​TGC​GTC​TGT​GCT​TG	TCC​ACA​AAG​GAC​GCA​CTT​GA
M-Keap1	GTG​GGC​CGC​CTC​ATC​TAC​AC	TGG​AGT​CAG​TGT​TGC​CAT​CC
M- Hmox1	GAG​TTC​TGC​TGA​GGC​TGA​TG	CCT​CTC​AGA​GGG​CAC​TGT​TC

### 2.8 Westen blot

Proteins were extracted from processed lung tissue and cell samples using RIPA lysis buffer (Beyotime, P0013C, China). Protein concentration was determined by the BCA method (Beyotime, P0011, China). All protein bands were separated on 10% SDS-PAGE gels (Yazyme, PG113, China). Subsequently, the proteins were transferred onto PVDF membranes. After blocking with 3% skim milk at room temperature for 90 min, the membranes were incubated with primary antibodies at 4 °C overnight (antibody details are provided in Table 2). The following day, the membranes were washed and incubated with an HRP-conjugated secondary antibody (Abcam, ab6789, United States). Proteins were visualized using a chemiluminescence system (Syngene, Affinity, United States). GAPDH was used as the loading control for normalization, and quantitative analysis was performed with ImageJ.

**TABLE 2:** The antibodies for Western blot.

Antibody	Company and item number	Dilution
GAPDH	Affinity, AF7021	1:5,000
VE-cadherin	Affinity, AF6265	1:1,000
VEGFR2	Affinity, AF6281	1:1,000
P-VEGFR2	Affinity, AF4426	1:1,000
Cleaved-caspase 9	Affinity, AF5240	1:1,000
Caspase 9	Affinity, AF6348	1:1,000
Cleaved-caspase 3	Affinity, AF7022	1:1,000
Caspase 3	Affinity, AF6311	1:1,000
Bax	Affinity, AF0120	1:1,000
Bcl2	Santa Cruz biotechnology, sc-56015	1:1,000
Keap1	Affinity, AF5266	1:1,000
HO-1	Affinity, AF5393	1:1,000
JNK	Affinity, AF6318	1:1,000
P-JNK	Affinity, AF3318	1:1,000
ERK	Cell signaling technology, 9102S	1:1,000
P-ERK	Affinity, AF1015	1:1,000
p38	Cell signaling technology, 9212S	1:1,000
P-p38	Cell signaling technology, 9211S	1:1,000
AKT	Affinity, AF0836	1:1,000
P-AKT	Affinity, AF0016	1:1,000
PI3K	Affinity, AF6241	1:1,000
P-PI3K	Affinity, AF3242	1:1,000
Ki67	Affinity, AF0198	1:1,000

### 2.9 Immunohistochemistry

After deparaffinization and rehydration, tissue sections were completely immersed in 3% hydrogen peroxide solution (MKbio, MM0750, China) to inactivate endogenous peroxidase and incubated at room temperature for 10 min. Antigen retrieval was performed using microwave-assisted heating by immersing the sections in antigen retrieval solution (Fuzhou Maixin Biotech Co., Ltd, MVS-0101, China). Sections were blocked with 5% BSA (Solarbio, A8020, China) at 37 °C for 1 h and subsequently incubated with primary antibodies overnight at 4 °C. Staining was carried out using an immunohistochemistry kit (ABclonal, RK05872). The expression of α-SMA (Santa, sc-32251, 1:200) was detected to assess vascular remodeling. Images of tissue sections were acquired under an upright microscope, and the ratio of stained area to total area was analyzed using ImageJ.

### 2.10 Wound healing assay

MOVAS cells were seeded into 12-well plates. Before inflicting the wound, the cells were fully confluent. A scratch was made in the center of the culture well using a sterile 200 μL micropipette tip. The wounds were observed using an inverted optical microscope, and multiple images were obtained at areas flanking the intersections of the wound and the marker lines after the scratch at 0, 6, 24, and 48 h. The images were analyzed using ImageJ software.

### 2.11 Detection of ROS by flow cytometry

After drug stimulation, the ROS probe was loaded and cells were collected according to the instructions of the Reactive Oxygen Species Assay Kit (leigen, TO0205, China). Following drug treatment, the ROS probe was diluted with serum-free medium. The drug-containing medium was removed, and the diluted probe was added to the cells, which were then incubated in the dark at 37 °C for 30 min. After incubation, the cells were collected into centrifuge tubes, washed three times with PBS, and analyzed by either flow cytometry or fluorescence microplate reader.

### 2.12 Annexin V-FITC/PI flow cytometry

After drug stimulation, cells were collected and processed according to the instructions of the Apoptosis Detection Kit (Beijing Solarbio Science and Technology Co., Ltd., CA1020, China). For adherent cells, the culture medium was first collected into centrifuge tubes, and trypsin (without EDTA) was added to detach the cells. The previously collected medium was then added back into the corresponding wells, and the cells were harvested into centrifuge tubes. Samples were centrifuged at 1,000 rpm for 5 min, and the supernatant was carefully aspirated. Cells were resuspended in 1 mL of pre-cooled PBS at 4 °C and centrifuged again to remove the supernatant. Subsequently, cells were resuspended in binding buffer and adjusted to a concentration of 1–5 × 10^6^ cells/mL. A 100 μL aliquot of the cell suspension was transferred to a 5 mL flow cytometry tube, followed by addition of 5 μL Annexin V-FITC and thorough mixing. The mixture was incubated at room temperature for 5 min in the dark. Then, 5 μL of propidium iodide (PI) was added, followed by 400 μL of PBS. Fluorescence channels for FITC and PI were selected, and flow cytometry analysis was performed immediately.

### 2.13 Measurement of SOD content

Samples were prepared according to the instructions of the SOD assay kit (Nanjing Jiancheng Bioengineering Institute, A001-3, China). All procedures were performed following the manufacturer’s protocol. Absorbance was measured at 450 nm using a microplate reader.

### 2.14 Measurement of GSH content

Samples were prepared accordance to the instructions of the GSH assay kit (Nanjing Jiancheng Bioengineering Institute, A006-2-1, China). All procedures were performed following the manufacturer’s protocol. Absorbance was measured at 405 nm using a microplate reader.

### 2.15 Endothelial cell tube formation assay

Matrigel (YEASEN, 40186ES10, China) was thawed overnight at 4 °C. Pre-cooled 24-well plates were coated with 100 μL of Matrigel per well and incubated at 37 °C for 1 h to allow polymerization. After solidification, cells were resuspended in drug-containing medium and seeded uniformly into the 24-well plates at approximately 1.2 × 10^5^ cells per well. Tube formation was observed every 4 h. Images were processed using ImageJ.

### 2.16 Statistical analysis

All experimental data, which were shown as Mean ± SEM, were conducted and analyzed through Prism 9.0. The difference upon above two groups was analysed by one-way analysis of variance ANOVA with Tukey’s *post hoc* multiple comparison test. *P* < 0.05 was statistically significant.

## 3 Results

### 3.1 Tacrolimus ameliorates bleomycin-induced pulmonary hypertension and fibrosis in rats

To verify the alleviating effect of Tacrolimus on fibrosis-associated pulmonary hypertension, we established a rat model of pulmonary fibrosis-associated pulmonary hypertension by intratracheal injection of bleomycin (Figure 1A) and assessed relevant parameters. Measurements via right heart catheterization revealed that bleomycin induction significantly increased right ventricular systolic pressure (RVSP) in the rats. Compared with the model group, rats treated with Tacrolimus showed a significant reduction in RVSP (Figures 1B,C). Further results from echocardiography and cardiac tissue sections demonstrated that Tacrolimus treatment markedly improved right heart function (Figures 1D,E) and significantly reduced right ventricular wall thickness (Figures 1F,G). These findings indicate that bleomycin effectively induces elevated pulmonary arterial pressure and increased right ventricular pressure load in rats, while Tacrolimus significantly alleviates bleomycin-induced pulmonary hypertension and right heart pressure load. Lung tissue section staining showed that bleomycin stimulation significantly increased pulmonary fibrosis, while Tacrolimus effectively attenuated these fibrotic changes induced by bleomycin. Consistent results were also obtained from Masson’s trichrome and Sirius red staining (Figures 1H–K). In addition, analysis of fibrosis-related gene expression indicated that Tacrolimus significantly downregulated the expression of fibrosis markers (Figures 1L,M).

**FIGURE 1:**
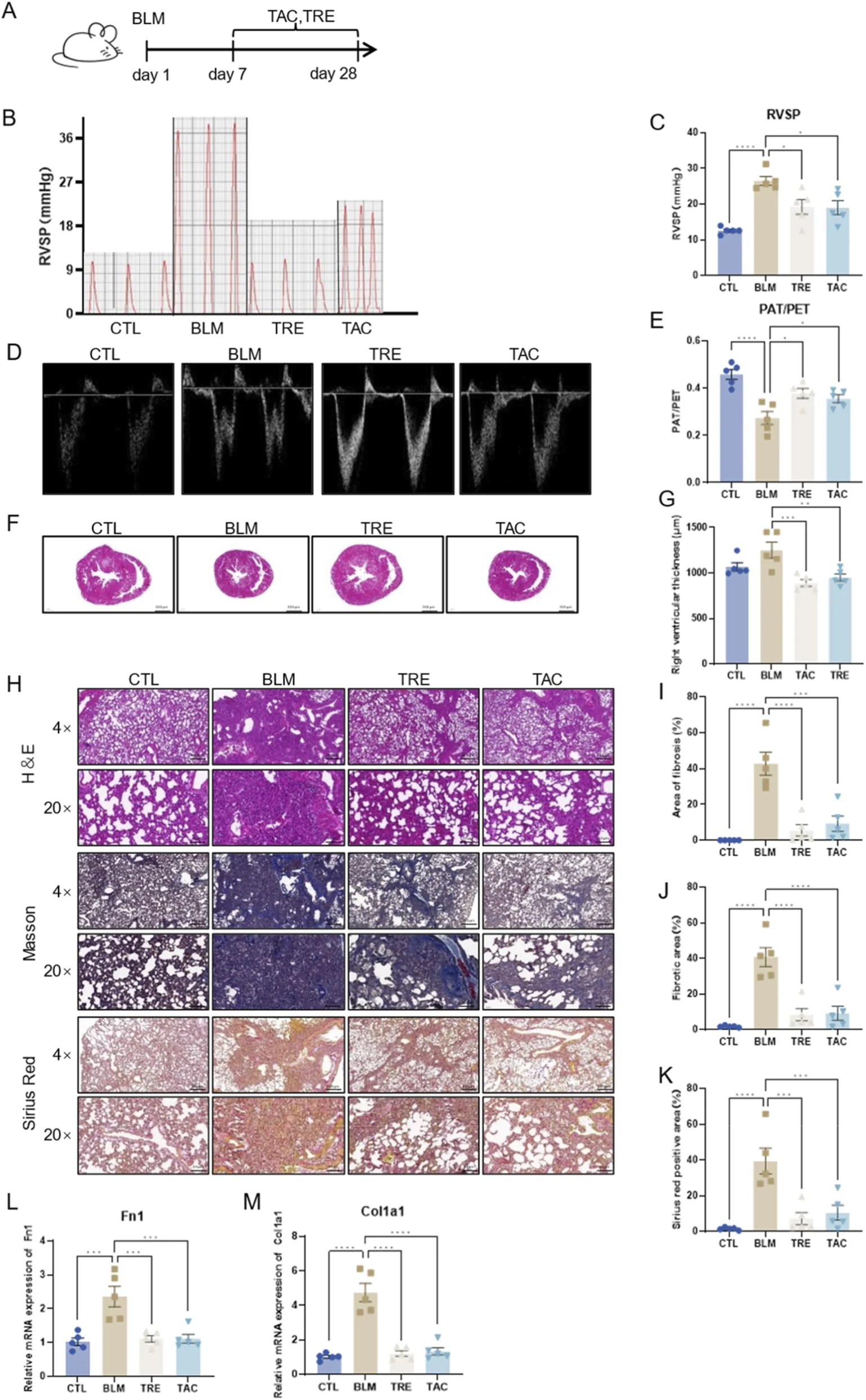
Tacrolimus alleviates bleomycin-induced pulmonary hypertension and pulmonary fibrosis in rats. **(A)** Schematic diagram of bleomycin-induced pulmonary hypertension associated with pulmonary fibrosis model. **(B)** Representative images of right ventricular systolic pressure. **(C)** Statistical analysis of right ventricular systolic pressure (RVSP, mmHg). **(D)** Representative images of echocardiographic. **(E)** Statistical analysis of PAT/PET (%). **(F)** Representative cardiac sections with H&E staining (×2 magnification, scale bar = 500 μm). **(G)** Statistical analysis of right ventricular wall thickness (μm). **(H)** Representative lung sections with H&E staining (4× magnification, scale bar = 200 μm and 20× magnification, scale bar = 40 μm), Masson’s trichrome staining (4× magnification, scale bar = 200 μm and 20× magnification, scale bar = 40 μm), and Sirius red staining (4× magnification, scale bar = 200 μm and 20× magnification, scale bar = 40 μm). **(I)** Percentage of lung fibrosis area in each group of rats. **(J)** Quantification of collagen density in lung tissues on Masson’s trichrome-stained images. **(K)** Quantification of collagen density in lung tissues on Sirius red-stained images. **(L)** Statistical analysis of Fn1 in lung tissues by QRT-PCR. **(M)** Statistical analysis of Col1a1 in lung tissues by QRT-PCR. We applied GAPDH as normalization for all the genes. The experimental data were presented as Mean ± SEM, and analyzed using one-way ANOVA with Tukey’s *post hoc* multiple comparison test. We used * to represent the difference between the BLM and other groups (*P < 0.05; **P < 0.01; ***P < 0.001; ****P < 0.0001).

### 3.2 Tacrolimus ameliorates vascular remodeling in bleomycin-induced rat models

Vascular remodeling is a key pathological feature of pulmonary hypertension. H&E staining of lung tissues revealed marked remodeling of microvessels in bleomycin-induced rat lungs, with significant thickening of the vascular wall (Figures 2A,B). Immunohistochemical staining for the vascular smooth muscle cell marker α-SMA further confirmed this observation (Figures 2C,D). After bleomycin induction, the muscularization of vessels was notably enhanced; however, treatment with Tacrolimus improved both vascular wall thickness and the degree of muscularization. These findings were further supported by the expression of angiogenesis-related proteins in lung tissues (Figures 2E–G).

**FIGURE 2:**
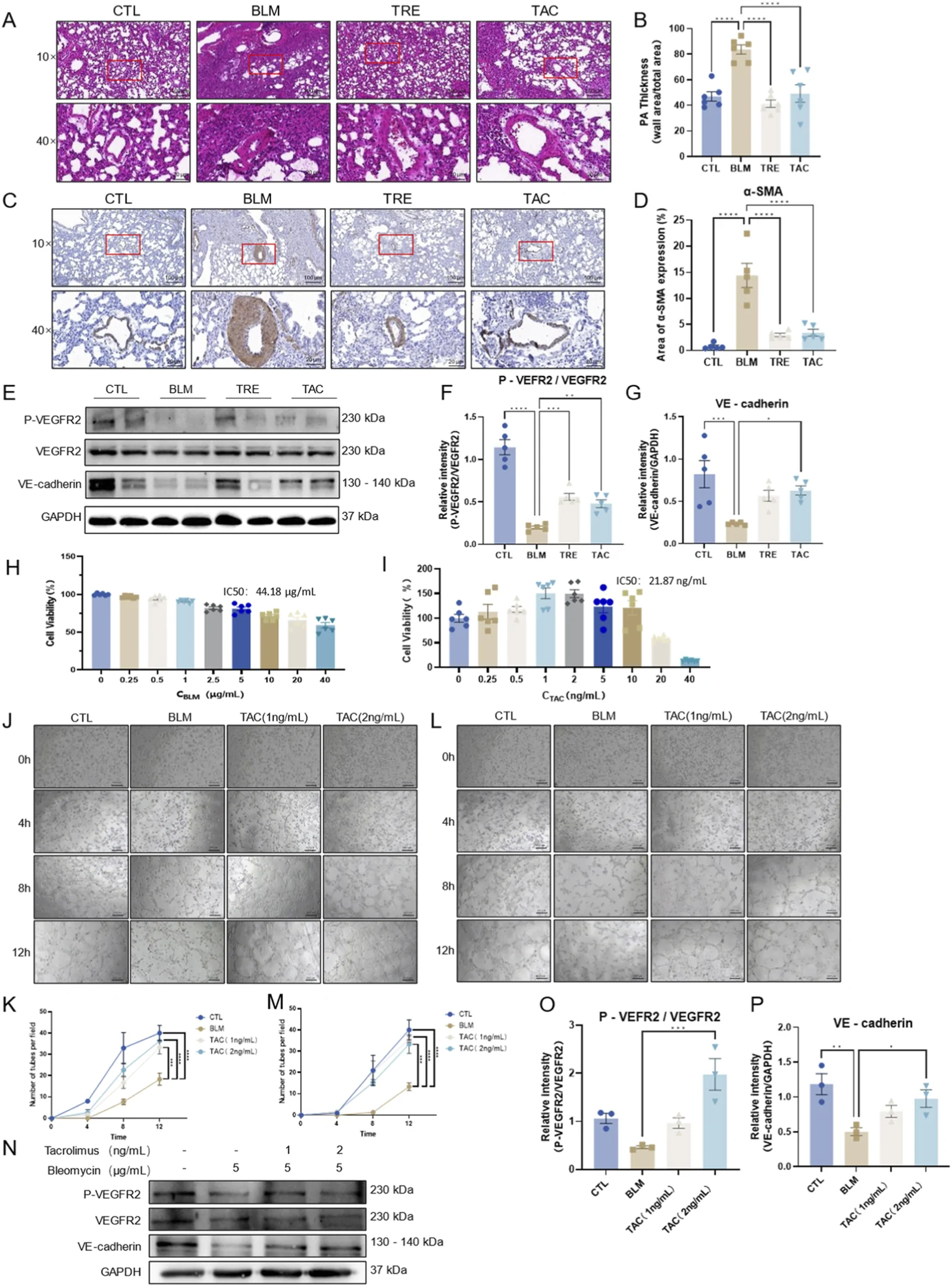
Tacrolimus ameliorates vascular remodeling in bleomycin-induced rat models. **(A)** Representative lung sections with H&E staining (10× magnification, scale bar = 100 μm and 40× magnification, scale bar = 20 μm). **(B)** Statistical analysis of vascular wall thickness. **(C)** Representative immunohistochemical staining images of α-SMA in lung tissue of each group of rats (10× magnification, scale bar = 100 μm and 40× magnification, scale bar = 20 μm). **(D)** Statistical analysis of α-SMA positive areas in lung tissue under 10× magnification (%). **(E)** Representative images of P-VEGFR2, VEGFR2, and VE-cad herin in lung tissues by Western blot. **(F)** Statistical analysis of P-VEGFR2/VEGFR2 in lung tissues by Western blot. **(G)** Statistical analysis of VE-cadherin in lung tissues by Western blot. **(H)** C166 cells were exposed to BLM at concentrations ranging from 0.25 μg/mL to 40 μg/mL for 24 h, and cell viability was assessed using the CCK-8 assay. **(I)** C166 cells were exposed to tacrolimus at concentrations ranging from 0.25 to 40 ng/mL for 24 h, and cell viability was assessed using the CCK-8 assay. **(J)** Representative images of C166 cells forming tubes *in vitro*. **(K)** Statistical analysis of C166 cells forming tubes *in vitro*. **(L)** Representative images of PAECs cells forming tubes *in vitro*. **(M)** Statistical analysis of PAECs cells forming tubes *in vitro*. **(N)** Representative images of P-VEGFR2, VEGFR2, and VE-cadherin in C166 by Western blot. **(O)** Statistical analysis of P-VEGFR2/VEGFR2 in C166 by Western blot. **(P)** Statistical analysis of VE-cadherin in C166 by Western blot. The experimental data were presented as Mean ± SEM, and analyzed using one-way ANOVA with Tukey’s *post hoc* multiple comparison test. We used * to represent the difference between the BLM and other groups (*P < 0.05; **P < 0.01; ***P < 0.001; ****P < 0.0001).

Vascular endothelial cells play a central role in the vascular remodeling process associated with pulmonary fibrosis-related pulmonary hypertension. To further evaluate the effect of Tacrolimus on endothelial cells during disease progression, we conducted *in vitro* experiments using mouse vascular endothelial cells (C166). First, the effects of Tacrolimus (0.25–40 ng/mL) and bleomycin (0.25–40 μg/mL) on C166 cell viability were assessed using the CCK-8 assay (Figures 2H,I). Based on the literature and our experimental results, we selected 5 μg/mL bleomycin and 1 ng/mL or 2 ng/mL Tacrolimus for subsequent *in vitro* studies. In an *in vitro* endothelial tube formation assay, bleomycin treatment significantly inhibited the formation of tubularstructures, while Tacrolimus restored the angiogenic capacity of C1 66 cells (Figures 2J,K). The same effect was observed in primary rat pulmonary arterial endothelial cells (PAECs) (Figures 2L,M). Further analysis of angiogenesis-related proteins *in vitro* showed that bleomycin significantly suppressed endothelial-related phosphorylated VEGFR2 (P-VEGFR2) and VE-cadherin expression. In contrast, Tacrolimus reversed this inhibitory effect, suggesting that it may improve bleomycin-induced vascular remodeling by promoting endothelial cell-mediated angiogenesis (Figures 2N–P).

### 3.3 Tacrolimus mitigates bleomycin-induced endothelial cell apoptosis

Endothelial cell apoptosis is a significant form of cell death in the pathogenesis of IPF-PH (Jin and Choi, 2012). Within the apoptotic pathway, the balance between the Bcl-2 family proteins Bax (pro-apoptotic) and Bcl-2 (anti-apoptotic) critically influences the progression of apoptosis (Yang et al., 2008). Analysis of apoptosis-related proteins and gene expression in lung tissue showed that after BLM induction, both apoptotic-related genes and proteins were significantly upregulated. Following Tacrolimus treatment, however, the protein level of Bax decreased markedly (Figures 3A,B), and its gene expression was correspondingly reduced (Figure 3D). Conversely, the expression of the anti-apoptotic protein Bcl-2 and its corresponding gene was significantly increased (Figures 3C,E). These results indicate that BLM induction triggered substantial apoptosis in lung tissue, which was markedly alleviated after drug administration.

**FIGURE 3:**
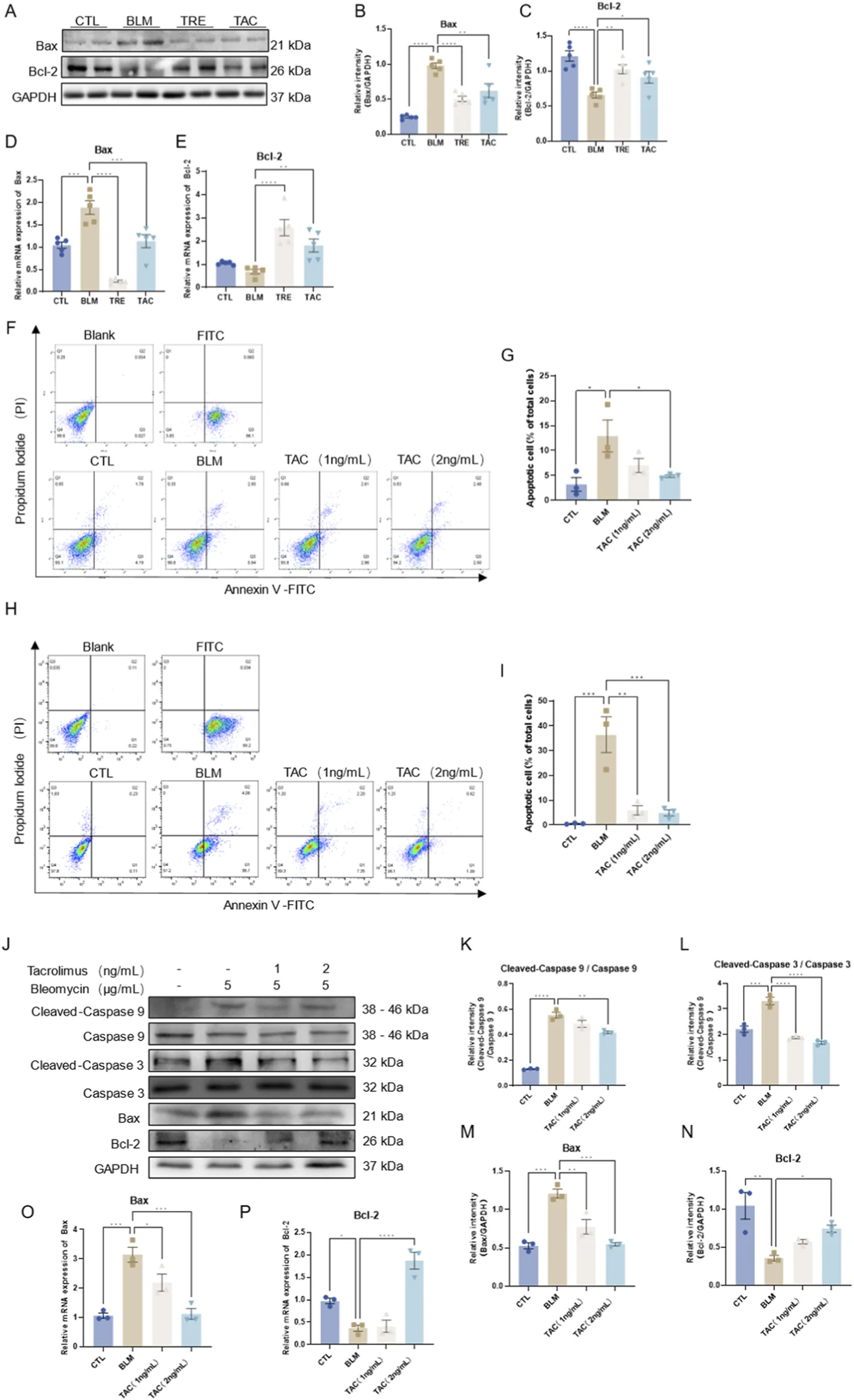
Tacrolimus mitigates bleomycin-Induced endothelial cell apoptosis. **(A)** Representative images of Bax and Bcl-2 in lung tissues by Western blot. **(B)** Statistical analysis of Bax in lung tissues by Western blot. **(C)** Statistical analysis of Bcl-2 in lung tissues by Western blot. **(D)** Statistical analysis of Bax in lung tissues by qRT-PCR. **(E)** Statistical analysis of Bcl-2 in lung tissues by qRT-PCR. **(F)** Representative images of apoptosis in PAECs cells measured by flow cytometry. **(G)** Statistical analysis of PAECs apoptotic cells (%). **(H)** Representative images of apoptosis in C166 cells measured by flow cytometry. **(I)** Statistical analysis of C166 apoptotic cells (%). **(J)** Representative images of Cleaved-Caspase 9, Caspase 9, Cleaved-Caspase 3, Caspase 3, Bax and Bcl-2 in C166 by Western blot. **(K)** Statistical analysis of Cleaved-Caspase 9/Caspase 9 in C166 by Western blot. **(L)** Statistical analysis of Cleaved-Caspase 3/Caspase 3 in C166 by Western blot. **(M)** Statistical analysis of Bax in C166 by Western blot. **(N)** Statistical analysis of Bcl-2 in C166 by Western blot. **(O)** Statistical analysis of Bax in C166 by qRT-PCR. **(P)** Statistical analysis of Bcl-2 in C166 by qRT-PCR. We applied GAPDH as normalization for all the genes. The experimental data were presented as Mean ± SEM, and analyzed using one-way ANOVA with Tukey’s *post hoc* multiple comparison test. We used * to represent the difference between the BLM and other groups (*P < 0.05; **P < 0.01; ***P < 0.001; ****P < 0.0001).

Further analysis of apoptosis at the cellular level was performed using Annexin V-FITC/PI flow cytometry. The results showed that bleomycin stimulation significantly induced apoptosis in PAECs cells, while Tacrolimus treatment effectively inhibited this process (Figures 3F,G). Similarly, Tacrolimus ameliorated bleomycin-induced apoptosis in C166 cells (Figures 3H,I). In the apoptotic pathway, the Caspase family plays a pivotal role. Cleaved Caspase-9 and Cleaved Caspase-3, the activated forms of Caspase-9 and Caspase-3, respectively, increase during apoptosis. After bleomycin stimulation of endothelial cells, protein levels of cleaved Caspase-9, cleaved Caspase-3 and Bax were elevated (Figures 3J–M), while expression of the anti-apoptotic protein Bcl-2 was significantly reduced (Figures 3J,N), consistent with gene expression results. Following Tacrolimus treatment, the levels of these related genes and proteins were restored (Figures 3O,P). These findings indicate that Tacrolimus can regulate apoptosis-related molecules, restore the balance between pro-apoptotic and anti-apoptotic signaling in endothelial cells under bleomycin-induced stress, and thereby attenuate endothelial cell apoptosis.

### 3.4 Tacrolimus attenuates oxidative stress in endothelial cells

In the progression of IPF-PH, sustained hypoxia and impaired ventilation within fibrotic areas stimulate the overproduction of reactive oxygen species (ROS) (Walters et al., 2008). This leads to oxidative stress in vascular endothelial cells, characterized by the downregulation of antioxidant enzymes such as superoxide dismutase (SOD) and antioxidant molecules including glutathione (GSH) (Chen et al., 2016). Persistent oxidative damage subsequently promotes endothelial apoptosis, disrupts vascular integrity, and accelerates vascular remodeling. Thus we measured the expression levels of SOD and GSH in lung tissues. The results showed that BLM induction significantly increased both SOD and GSH in lung tissue, and Tacrolimus attenuated these changes (Figures 4A,B). The Keap1-HO-1 pathway plays an important role in oxidative stress responses. Analysis of related genes and proteins in this pathway revealed that bleomycin upregulated Keap1 expression and downregulated HO-1 expression, indicating oxidative stress in lung tissue. Treatment with Tacrolimus reversed these changes (Figures 4C–G).

**FIGURE 4:**
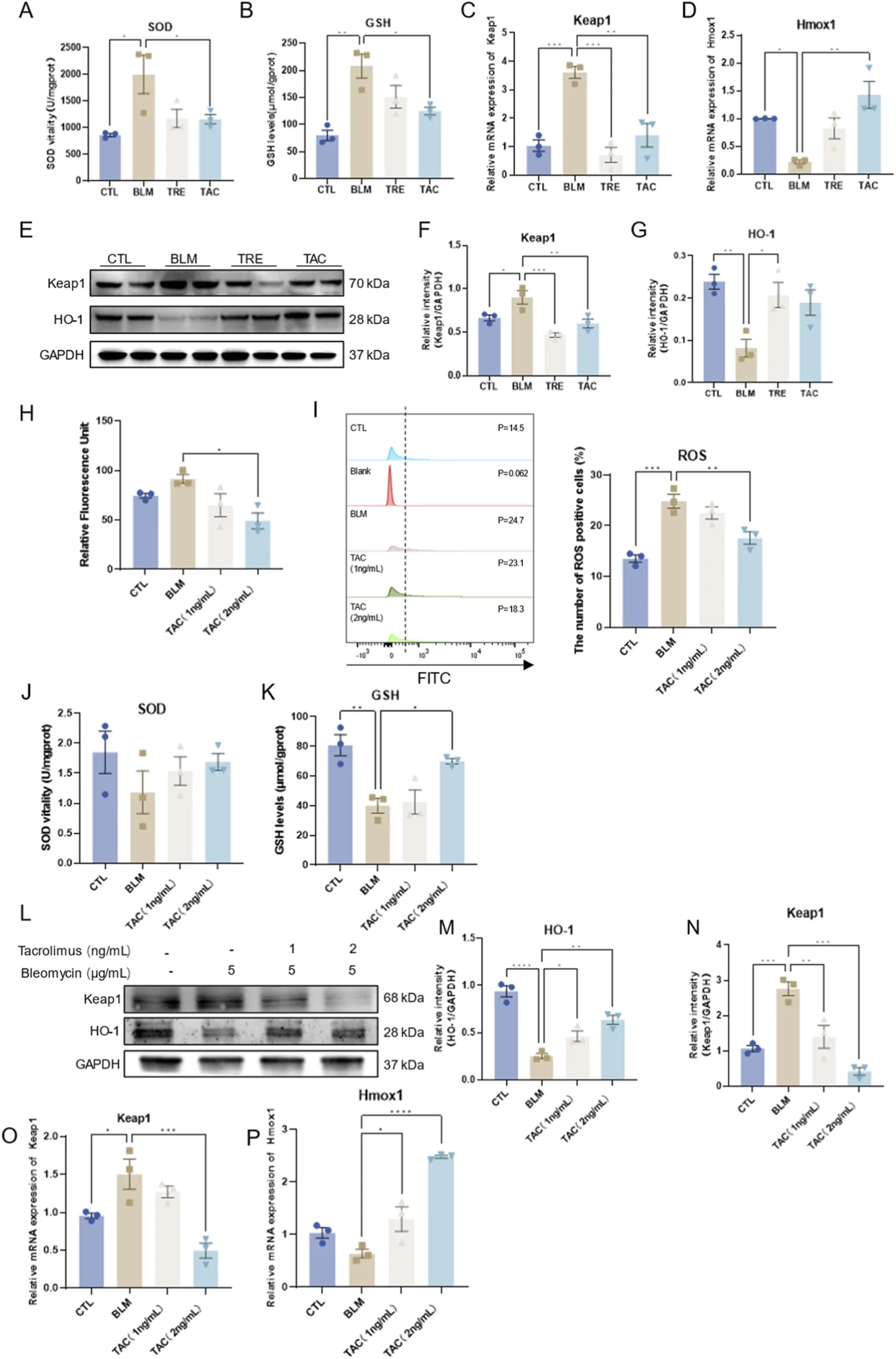
Tacrolimus attenuates oxidative stress in endothelial cells. **(A)** Statistical analysis of SOD vitality in lung tissue (U/mgprot). **(B)** Statistical analysis of GSH vitality in lung tissue (μmol/gprot). **(C)** Statistical analysis of Keap1 in lung tissues by qRT-PCR. **(D)** Statistical analysis of Hmox1 in lung tissues by qRT-PCR. **(E)** Representative images of Keap1 and HO-1 in lung tissues by Western blot. **(F)** Statistical analysis of Keap1 in lung tissues by Western blot. **(G)** Statistical analysis of HO-1 in lung tissues by Western blot. **(H)** ROS levels in PAECs were determined by DCFH-DA fluorescence using a microplate reader. **(I)** Representative images of ROS-positive cells detected by flow cytometry and statistical analysis of ROS-positive cells (%). **(J)** Statistical analysis of SOD vitality in C166 (U/mgprot). **(K)** Statistical analysis of GSH vitality in C166 (μmol/gprot). **(L)** Representative images of Keap1 and HO-1 in C166 by Western blot. **(M)** Statistical analysis of Keap1 in C166 by Western blot. **(N)** Statistical analysis of HO-1 in C166 by Western blot. **(O)** Statistical analysis of Keap1 in C166 by Western blot. **(P)** Statistical analysis of Hmox1 in C166 by qRT-PCR. We applied GAPDH as normalization for all the genes. The experimental data were presented as Mean ± SEM, and analyzed using one-way ANOVA with Tukey’s *post hoc* multiple comparison test. We used * to represent the difference between the BLM and other groups (*P < 0.05; **P < 0.01; ***P < 0.001; ****P < 0.0001).

Furthermore, using the DCFH-DA probe, we found that bleomycin significantly elevated ROS production in PAECs *in vitro*, while Tacrolimus effectively attenuated this oxidative stress (Figure 4H). Intracellular ROS levels in C166 cells after bleomycin stimulation were detected by flow cytometry. The results showed that bleomycin induced a significant increase in ROS in C166 cells, while Tacrolimus treatment markedly reduced intracellular ROS levels (Figure 4I). Similarly, both SOD and GSH levels were restored by Tacrolimus treatment, indicated that Tacrolimus could significantly enhance the antioxidant capacity of endothelial cells (Figures 4J,K). Further analysis of Keap1 and HO-1 related genes and proteins in cells demonstrated that Tacrolimus effectively modulated their expression (Figures 4L–P). These findings indicate that Tacrolimus alleviates oxidative stress in vascular endothelial cells via the Keap1-HO-1 pathway, thereby mitigating oxidative stress in lung tissue.

### 3.5 Tacrolimus suppresses the MAPK and PI3K/AKT pathways both *in vitro* and *in vivo*

Previous studies have demonstrated that oxidative stress can activate the MAPK signaling pathway, which in turn triggers caspase activation and promotes cellular apoptosis (Xiao et al., 2013). While PI3K/AKT signaling pathway serves as a critical regulator of cell survival and plays an important role in apoptosis (Wang et al., 2022). To further investigate the molecular mechanism by which Tacrolimus modulates the pathological phenotype of vascular endothelial cells in IPF-PH, we examined the MAPK and PI3K/AKT pathways in both cellular and animal models. Western blot results showed that BLM significantly activated both the MAPK and PI3K/AKT pathways. Following Tacrolimus intervention, the phosphorylation and expression levels of key proteins in the MAPK pathway (JNK, ERK, p38) and the PI3K/AKT pathway (PI3K, AKT) were significantly downregulated (Figures 5A–G). Consistent results were also observed in vascular endothelial cells. Collectively, these data suggest that Tacrolimus may exert its therapeutic effects by coordinately inhibiting the MAPK and PI3K/AKT signaling pathways (Figures 5H–N).

**FIGURE 5:**
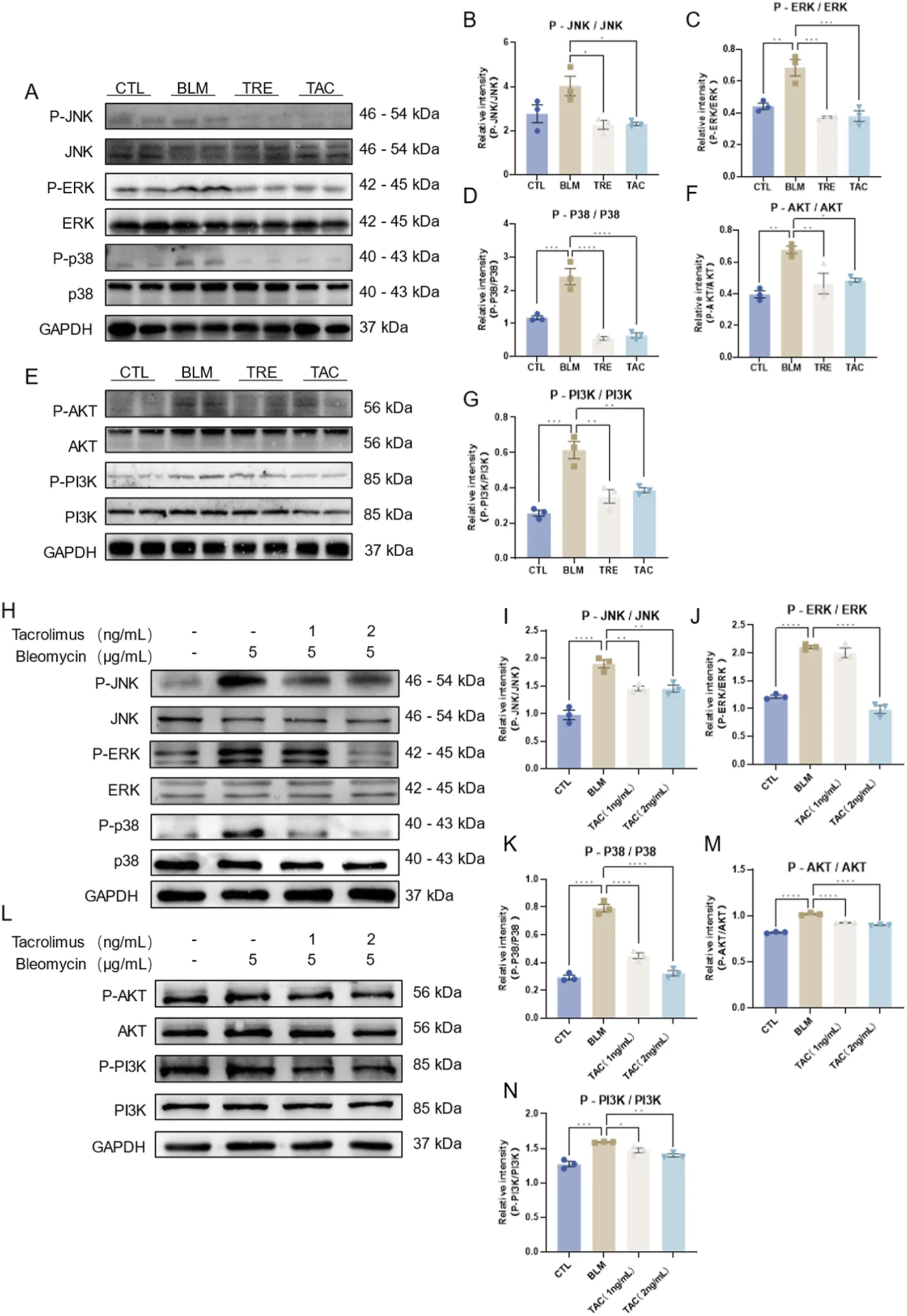
Tacrolimus inhibits the MAPK and PI3K/AKT pathways both *in vivo* and *in vitro*. **(A)** Representative images of P-JNK, JNK, P-ERK, ERK, P-p38, p38 in lung tissues by Western blot. **(B)** Statistical analysis of P-JNK/JNK in lung tissues by Western blot. **(C)** Statistical analysis of P-ERK/ERK in lung tissues by Western blot. **(D)** Statistical analysis of P-p38/p38 in lung tissues by Western blot. **(E)** Representative images of P-AKT, AKT, P-PI3K, PI3K in lung tissues by Western blot. **(F)** Statistical analysis of P-AKT/AKT in lung tissues by Western blot. **(G)** Statistical analysis of P-PI3K/PI3K in lung tissues by Western blot. **(H)** Representative images of P-JNK, JNK, P-ERK, ERK, P-p38, p38 in C166 by Western blot. **(I)** Statistical analysis of P-JNK/JNK in C166 by Western blot. **(J)** Statistical analysis of P-ERK/ERK in C166 by Western blot. **(K)** Statistical analysis of P-p38/p38 in C166 by Western blot. **(L)** Representative images of P-AKT, AKT, P-PI3K, PI3K in C166 by Western blot. **(M)** Statistical analysis of P-AKT/AKT in C166 by Western blot. **(N)** Statistical analysis of P-PI3K/PI3K in C166 by Western blot. The experimental data were presented as Mean ± SEM, and analyzed using one-way ANOVA with Tukey’s *post hoc* multiple comparison test. We used * to represent the difference between the BLM and other groups (*P < 0.05; **P < 0.01; ***P < 0.001; ****P < 0.0001).

### 3.6 Tacrolimus inhibits vascular smooth muscle cell proliferation *in vitro*

Another key phenotypic feature of vascular remodeling is the proliferation of smooth muscle cells (Ma et al., 2023). To explore the effect of Tacrolimus on smooth muscle cells, we stimulated mouse vascular smooth muscle cells (MOVAS) with different concentrations of TGF-β1. Results showed that TGF-β1 at a concentration of 2 ng/mL was sufficient to induce MOVAS cell proliferation (Figure 6A). Based on literature and preliminary experiments, 5 ng/mL TGF-β1 was selected for subsequent studies. Cytotoxicity assays indicated that the tested concentrations of Tacrolimus had no significant toxic effect on MOVAS cells (Figure 6B). The CCK-8 assay confirmed that TGF-β1 promoted cell proliferation, while Tacrolimus significantly inhibited this effect (Figure 6C). Consistent findings were obtained from the scratch wound healing assay (Figures 6D,E). Furthermore, detection of the proliferation-related protein Ki67 showed that Tacrolimus also suppressed its expression (Figures 6F,G). These results indicate that Tacrolimus effectively inhibits smooth muscle cell proliferation *in vitro*.

**FIGURE 6:**
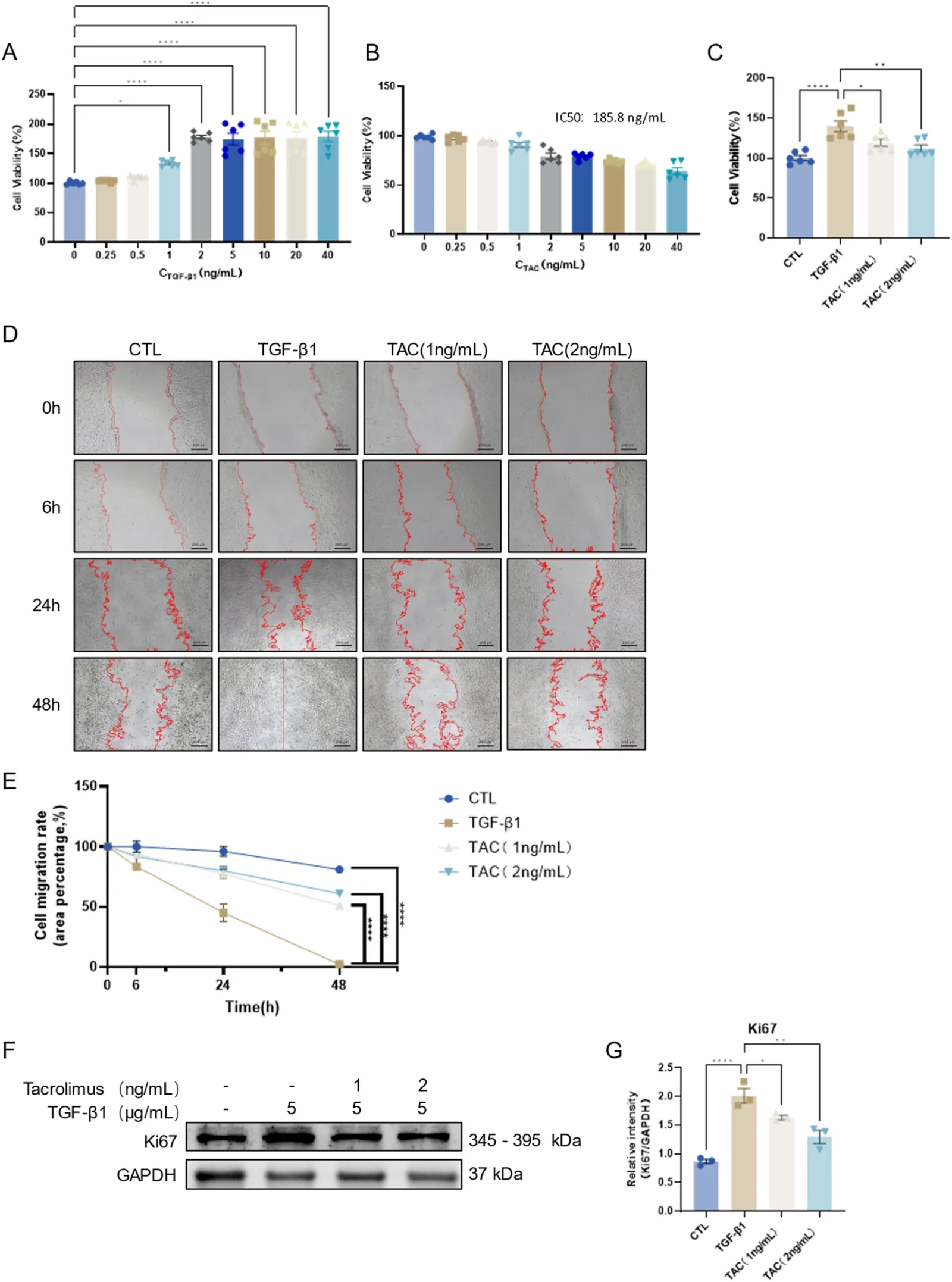
Tacrolimus inhibits vascular smooth muscle cell proliferation *in vitro*. **(A)** MOVAS cells were exposed to TGF-β1 at concentrations ranging from 0.25 to 40 ng/mL for 24 h, and cell viability was assessed using the CCK-8 assay. **(B)** MOVAS cells were exposed to tacrolimus at concentrations ranging from 0.25 to 40 ng/mL for 24 h, and cell viability was assessed using the CCK-8 assay. **(C)** Statistical analysis of MOVAS cells proliferation was assessed using the CCK-8 assay. **(D)** Representative images of MOVAS cells scratch wound healing at 0, 6, 24, and 48 h. **(E)** Statistical analysis of scratch wound healing at 0, 6, 24, and 48 h. **(F)** Representative images of Ki67 in MOVAS by Western blot. **(G)** Statistical analysis of Ki67 in MOVAS by Western blot. The experimental data were presented as Mean ± SEM, and analyzed using one-way ANOVA with Tukey’s *post hoc* multiple comparison test. We used * to represent the difference between the TGF-β1 and other groups (*P < 0.05; **P < 0.01; ***P < 0.001; ****P < 0.0001).

Integrating the above findings, we conclude that Tacrolimus alleviates bleomycin-induced pulmonary fibrosis-associated pulmonary hypertension in rats by regulating endothelial cell apoptosis and oxidative stress via the MAPK and PI3K/AKT signaling pathways. Tacrolimus effectively modulates vascular remodeling involving endothelial tight junctions and also significantly suppresses aberrant proliferation of mouse vascular smooth muscle cells induced by BLM (Figure 7).

**FIGURE 7:**
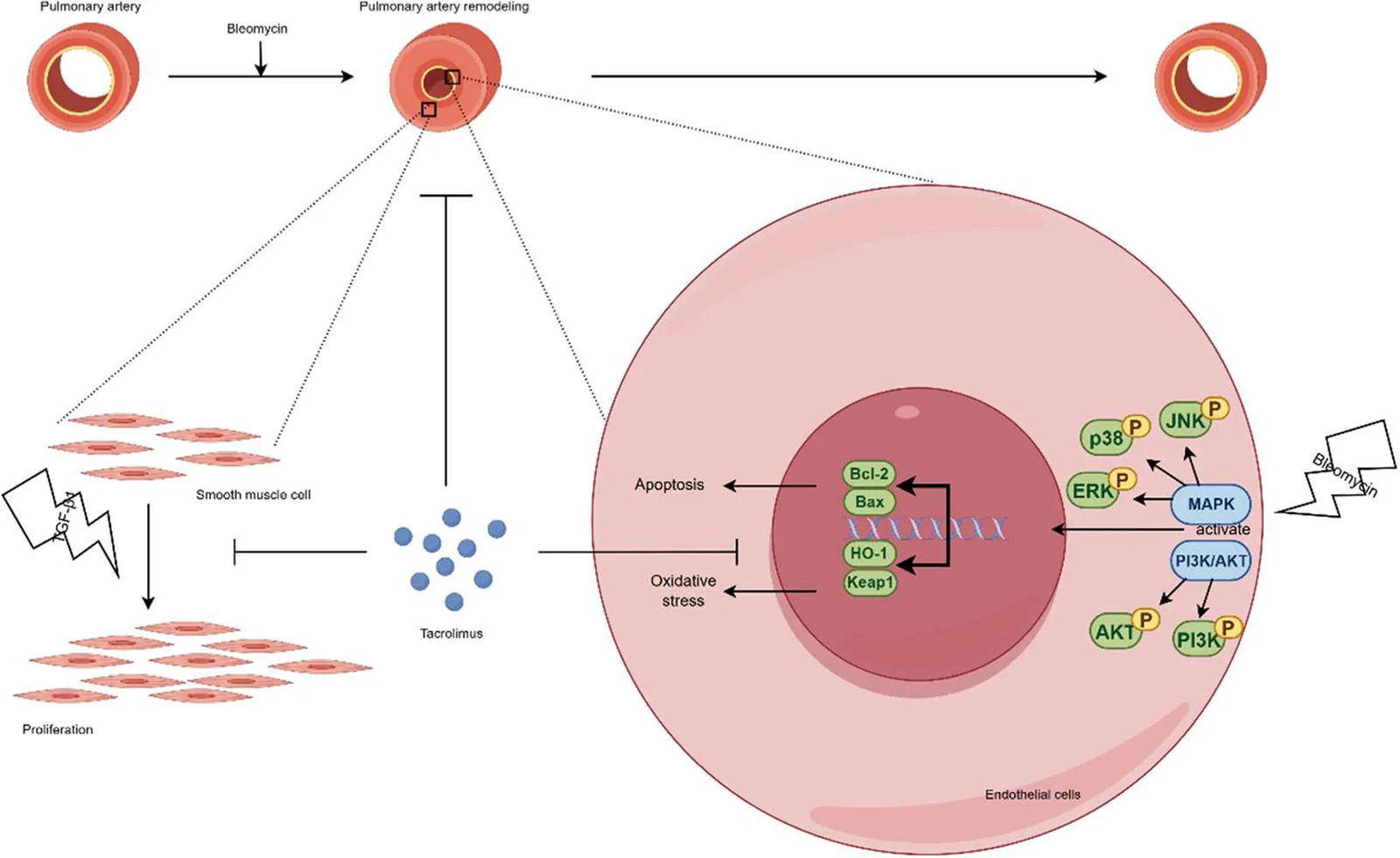
Mechanism of tacrolimus in attenuating bleomycin induced IPF-PH in rats.

## 4 Discussion

Idiopathic pulmonary fibrosis-associated pulmonary hypertension (IPF-PH) is a severe complication in the course of idiopathic pulmonary fibrosis. After developing PH, patients often present with symptoms such as dyspnea, exertional breathlessness, and dry cough. These symptoms not only significantly impair quality of life and exercise tolerance but also accelerate the decline in lung function and markedly shorten survival. The prognosis of this complication is extremely poor, ultimately leading to right heart failure and even death (Rajagopal et al., 2021). Its pathogenesis is generally attributed to compression of pulmonary arteries by lung scar tissue, which increases pulmonary vascular resistance and subsequently elevates pulmonary arterial pressure and pulmonary vascular resistance (George and Mitchell, 2019).

Currently, clinical management of both IPF and PH remains limited. First-line anti-fibrotic agents for IPF, such as nintedanib and pirfenidone, can slow the progression of lung function decline but offer limited benefit for established PH (Romana Stornelli et al., 2021). Meanwhile, targeted therapies for PH, including endothelin receptor antagonists, PDE5 inhibitors, and prostacyclin analogs, lack sufficient evidence regarding efficacy and safety in IPF-PH patients and fail to fundamentally reverse pulmonary vascular remodeling. Therefore, there remains an unmet clinical need for drugs that can simultaneously target both fibrosis and pulmonary hypertension by acting on key pathological pathways.

Tacrolimus, also known as FK506, is a macrolide immunosuppressant produced by fermentation of *Streptomyces* species and was originally developed by Astellas Pharma Inc. in Japan (Ordóñez-Robles et al., 2018). It specifically binds to the intracellular FK506-binding protein 12 (FKBP-12), and the resulting complex inhibits the activity of calcineurin. Calcineurin is a key enzyme in the T-cell receptor signaling pathway (Williams and Hong, 2019). Its inhibition blocks the dephosphorylation and nuclear translocation of nuclear factor of activated T cells (NFAT), thereby effectively suppressing T-cell activation and the gene transcription and release of various cytokines, such as IL-2 and IFN-γ (Broen and van Laar, 2020). This leads to potent immunosuppressive effects. Based on this mechanism, the classic clinical applications of tacrolimus have primarily focused on preventing rejection following organ transplantation and on treating certain autoimmune diseases (Kandikattu and Mishra, 2018). However, recent studies suggest that the effects of tacrolimus extend beyond immunosuppression. A number of preclinical investigations have demonstrated that tacrolimus exhibits significant therapeutic potential in various animal models of pulmonary arterial hypertension (Sommer et al., 2019). Its mechanisms of action likely involve multiple pharmacological activities independent of immunosuppression, including but not limited to: Anti-proliferative effects: Direct inhibition of abnormal proliferation and migration of pulmonary arterial smooth muscle cells and fibroblasts, thereby counteracting vascular remodeling (Sommer et al., 2019). Anti-inflammatory effects (Liu et al., 2024). Reduction of perivascular inflammatory cell infiltration through pathways such as inhibition of NF-κB signaling. Endothelial protection: Potential improvement of vascular endothelial dysfunction by modulating eNOS activity, reducing oxidative stress, and suppressing endothelial-to-mesenchymal transition (EndMT).

Currently, preclinical research on tacrolimus in the field of pulmonary hypertension is primarily based on conventional models of PH, such as those induced by monocrotaline or chronic hypoxia in rats (Krstic et al., 2024; Sztuka and Jasińska-Stroschein, 2017). The core pathological features of these models focus on structural and functional abnormalities within the pulmonary vasculature itself, manifested as abnormal proliferation and migration of pulmonary arterial smooth muscle cells, endothelial dysfunction, local inflammatory cell infiltration, and progressive pulmonary vascular remodeling. It is noteworthy that in these models, the lung parenchyma generally remains relatively intact, with pathological changes largely confined to the vascular system.

In contrast, the bleomycin-induced rat model of pulmonary fibrosis-associated pulmonary hypertension represents a more complex, composite disease model (Santos-Ribeiro et al., 2023). This model involves the simultaneous and intertwined progression of two key pathological processes: first, progressive interstitial fibrosis of the lung, characterized by widespread destruction of alveolar architecture, excessive deposition of extracellular matrix, and substantial accumulation of collagen; and second, the concomitant development of pulmonary hypertension. In this model, the mechanisms underlying pulmonary hypertension are more multifactorial (Jarman et al., 2014). They include not only the mechanical compression and traction exerted by fibrotic lung tissue on surrounding microvessels and the resulting local hypoxic microenvironment, but also the sustained activation of inflammatory responses and aberrant proliferative signaling pathways-such as TGF-β-shared between pulmonary fibrosis and pulmonary hypertension. This study confirms that tacrolimus effectively alleviates disease progression in a bleomycin-induced rat model of IPF-associated pulmonary hypertension, as demonstrated by reduced right ventricular systolic pressure, improved right heart function, and attenuated pulmonary fibrosis. This therapeutic effect is mediated through actions on the vascular system and amelioration of pulmonary vascular remodeling. Mechanistically, tacrolimus exerts a direct protective effect on vascular endothelial cells, mitigating their apoptosis and oxidative stress, and restores their angiogenic capacity *in vitro*. These effects involve the modulation of the MAPK and PI3K/AKT signaling pathways. Concurrently, tacrolimus significantly inhibits the proliferation of vascular smooth muscle cells. These findings suggest that tacrolimus may ameliorate the pathological progression of IPF-PH by multi-targeted improvement of pulmonary vascular structure and function.

Compared with previous studies on tacrolimus in pulmonary fibrosis, the present study provides new mechanistic and therapeutic insights. The dose of tacrolimus used here was selected based on that reported in the monocrotaline-induced pulmonary hypertension model, which is considerably lower than the doses commonly used in IPF studies. Our results demonstrate that this lower dose can also attenuate the degree of fibrosis. Moreover, whereas previous studies on tacrolimus in IPF have mainly focused on its immunomodulatory effects, the present work investigates tacrolimus from a vascular perspective, with particular emphasis on endothelial cells, smooth muscle cells, and pulmonary vascular remodeling in IPF-PH. These findings therefore extend beyond previous observations and suggest that low-dose tacrolimus may confer benefits that are not solely attributable to immunosuppression, providing new insights into its potential role in IPF-PH-associated pulmonary vascular remodeling.

Compared with current prostacyclin analogs used for PH, such as treprostinil, tacrolimus demonstrates distinct mechanisms of action. Treprostinil primarily reduces pulmonary arterial pressure through potent vasodilation and inhibition of platelet aggregation, often requiring continuous subcutaneous or intravenous administration, which entails high costs and inconvenience (Chaudhary et al., 2018). In contrast, tacrolimus in this study exhibited direct inhibition of smooth muscle proliferation, alleviation of endothelial injury, and promotion of vascular normalization, suggesting its potential advantage in reversing pulmonary vascular remodeling. Furthermore, its injection offers greater convenience.

Placing our findings in the context of the current therapeutic landscape for pulmonary fibrosis helps define the distinct position of tacrolimus as a novel experimental agent. At present, standard antifibrotic therapy for IPF relies mainly on pirfenidone and nintedanib. The former acts by inhibiting TGF-β and other fibrogenic mediators, while the latter is a multi-target tyrosine kinase inhibitor that blocks VEGF, FGF and PDGF receptors (Naqvi et al., 2024). Although both drugs slow the decline in forced vital capacity (FVC), they do not reverse established fibrosis, and their efficacy in coexisting pulmonary hypertension (PH) is limited–some studies even suggest that nintedanib may be associated with PH progression in IPF-PH patients (Lacedonia et al., 2022; Romana Stornelli et al., 2021). In recent years, the treatment landscape for IPF and progressive pulmonary fibrosis (PPF) has been rapidly reshaped by the emergence of novel agents. Among the most representative is nerandomilast, an oral preferential phosphodiesterase 4B (PDE4B) inhibitor that exerts both anti-inflammatory and antifibrotic effects by elevating intracellular cAMP levels. This drug has recently received FDA approval for IPF (Naqvi et al., 2024; D'Agnano et al., 2024), (Carriera et al., 2026). In addition, several other agents are in late-stage clinical development, including the LPA1 receptor antagonist admlparant (BMS-986278), the ROCK2 inhibitor zelasudil (RXC007), and the TNIK inhibitor rentosertib (ISM001-055), which was discovered using a generative artificial intelligence platform (Carriera et al., 2026). In contrast to these agents, which primarily target epithelial-mesenchymal transition (EMT) and fibroblast activation, tacrolimus in our study exhibited a prominent protective effect on the pulmonary vasculature. The pathogenesis of IPF-PH involves not only mechanical compression of vessels by fibrotic tissue, but also a vicious feed-forward loop driven by endothelial injury, oxidative stress and aberrant smooth muscle cell proliferation (Rajagopal et al., 2021; Barratt and Millar, 2014). Our data indicate that tacrolimus attenuates both pulmonary fibrosis and PH through inhibition of the MAPK and PI3K/AKT pathways, acting from a “vascular-centric” perspective. This mechanism is fundamentally different from the purely “antifibrotic” or “mono-vasodilatory” approaches (e.g., inhaled treprostinil) of current mainstream therapies. Therefore, tacrolimus may offer a novel therapeutic strategy centred on reversal of pulmonary vascular remodelling for the particularly challenging IPF-PH subtype.

Notably, there is growing interest in inhalation drug delivery for antifibrotic agents, aiming to maximise local lung concentrations while minimising systemic side effects. Inhaled pirfenidone (AP01) showed favourable lung function stabilisation and very low systemic exposure in a phase Ib trial; inhaled treprostinil has been approved for PH-ILD; and inhaled siRNA formulations targeting MMP7 and HSP47 have entered early-phase clinical studies (D'Agnano et al., 2024). This trend highlights that optimising drug delivery is as important as discovering new targets.

Although our study used subcutaneous injection of tacrolimus, which was effective in the animal model, systemic immunosuppression (nephrotoxicity, hyperglycaemia, increased infection risk) remains a major barrier to clinical translation. However, recent evidence suggests that the antifibrotic effects of tacrolimus are partly independent of its classical calcineurin/NFAT immunosuppressive pathway, particularly its direct actions on endothelial cells and fibroblasts (Liu et al., 2022; Liu et al., 2024). Therefore, we propose that tacrolimus is well suited for development as an inhaled formulation. Nebulised delivery directly to the lungs and pulmonary vasculature could theoretically lower blood drug concentrations, avoiding systemic side effects while enhancing local “vascular remodelling-reversing” effects. This may open a novel translational pathway for tacrolimus in IPF-PH.

This study has certain limitations. First, although we focused on exploring the non-immunomodulatory effects of tacrolimus in the IPF-PH model, tacrolimus itself is a classical immunosuppressant, and its potential immunomodulatory mechanisms involved in the development and progression of IPF-PH were not extensively investigated in this work. Second, our analysis primarily examined the overall effects of tacrolimus on signaling pathways such as MAPK and PI3K/AKT, without further identifying specific molecular targets or key effector proteins within these pathways. Third, for smooth muscle cells, we used the MOVAS cell line (mouse aortic smooth muscle cells) to examine the inhibitory effect of tacrolimus on proliferation; however, these results were obtained only in this cell line and have not been validated in primary rat pulmonary arterial smooth muscle cells or *in vivo*. This represents an additional limitation, and we plan to address it in future studies by extending our investigations to primary smooth muscle cells and, where feasible, complementary *in-vivo* experiments. Fourth, we acknowledge that reduced fibrosis may contribute to the improvement in pulmonary hemodynamics. While our *in vitro* experiments support a vascular-cell-associated action of tacrolimus, they do not establish that the *in vivo* hemodynamic benefit is independent of its antifibrotic effect. Fifth, we sincerely acknowledge that an appropriate positive control or reference compound was not included in the *in vitro* experiments. We fully agree that such a control would further strengthen the *in vitro* findings and allow a more direct comparison with the effects of tacrolimus. We regret this omission and have explicitly acknowledged it as a limitation of the present study. Future studies will include a well-established positive control or reference compound to better benchmark the effects of tacrolimus. Another limitation is that only a single concentration of tacrolimus was used in the animal experiments. Although this concentration was selected with reference to previous studies and showed a favorable effect, a systematic dose-response analysis was not performed. Therefore, the dose-dependent effects of tacrolimus could not be fully evaluated, and future studies with multiple doses are warranted. Future studies could also build on these findings to further dissect the role of tacrolimus in the immune microenvironment, and employ techniques such as gene editing and specific inhibitors to validate downstream key targets, thereby providing a more comprehensive understanding of its pharmacological mechanisms. In summary, tacrolimus demonstrates improving pulmonary vascular remodeling through endothelial protection in an IPF-PH model, modulation of MAPK and PI3K/AKT-related signaling pathways, and inhibition of smooth muscle proliferation. These properties position it as a potential therapeutic option distinct from existing treatments, particularly suitable for IPF-PH patients with significant vascular remodeling. Further preclinical safety evaluations and prospective clinical studies are warranted to clarify its efficacy and safety profile and to explore its potential for combination therapy with existing anti-fibrotic or vascular-targeted agents.

## Data Availability

The original contributions presented in the study are included in the article/supplementary material, further inquiries can be directed to the corresponding authors.
